# Assessment of bleeding risk in cancer patients treated with anticoagulants for venous thromboembolic events

**DOI:** 10.3389/fcvm.2023.1132156

**Published:** 2023-08-21

**Authors:** Géraldine Poénou, Emmanuel Tolédano, Hélène Helfer, Ludovic Plaisance, Florent Happe, Edouard Versini, Nevine Diab, Sadji Djennaoui, Isabelle Mahé

**Affiliations:** ^1^ Médecine Interne, Hôpital Louis Mourier, Assistance Publique Hôpitaux de Paris, Colombes, France; ^2^ Université de Paris Cité, Paris, France; ^3^ Unité Inserm UMR-S1140 Innovation Thérapeutique en Hémostase, Paris, France; ^4^ INNOVTE-FCRIN, CEDEX 2, Saint-Etienne, France

**Keywords:** cancer, venous thromboembolism, bleeding, risk assessment model (RAM), anticoagulant

## Abstract

**Introduction:**

Anticoagulant is the cornerstone of the management of VTE at the cost of a non-negligible risk of bleeding. Reliable and validated clinical tools to predict thromboembolic and hemorrhagic events are crucial for individualized decision-making for the type and duration of anticoagulant treatment. We evaluate the available risk models in real life cancer patients with VTE. The objectives of the study were to describe the bleeding of cancer patients with VTE and to evaluate the performance of the different bleeding models to predict the risk of bleeding during a 6-month follow-up.

**Materials and Methods:**

VTE-diagnosed patient's demographic and clinical characteristics, treatment regimens and outcomes for up to 6 months were collected. The primary endpoint was the occurrence of a major bleeding (MB) or a clinically relevant non major bleeding (CRNMB) event, categorized according to the ISTH criteria.

**Results:**

During the 6-months follow-up period, 26 out of 110 included patients (26.7%) experienced a bleeding event, with 3 recurrences of bleeding. Out of the 29 bleeding events, 19 events were CRNMB and 10 MB. One patient died because of a MB. Bleeding occurred in 27 % of the patients treated with DOACs and 22% of the patients treated with LMWH. Most of the bleedings were gastrointestinal (9 events, 31%); 26.9% of the bleedings occurred in patient with colorectal cancer and 19.6% in patients with lung cancer. In our cohort, none of the 10 RAMs used in our study were able to distinguish cancer patients with a low risk of bleeding, from all bleeding or non-bleeding patients. The Nieto et al. RAM had the best overall performance (C-statistic = 0.730, 95% CI (0.619–0.840)). However, it classified 1 out of 5 patients with major bleeding in the low risk of bleeding group. The rest of the RAMs showed a suboptimal result, with a range of C-statistic between 0.489, 95%CI (0.360–0.617)) and 0.532, 95%CI (0.406–0.658)).

**Conclusions:**

The management of CAT patients is challenging due to a higher risk of both recurrent VTE and bleeding events, as compared with non-cancer patients with VTE. None of the existing RAMs was able to consistently identify patients with risk of anticoagulant associated bleeding events.

## 1. Introduction

Venous thromboembolism (VTE) can occur in two forms: deep vein thrombosis (DVT) and pulmonary embolism (PE). Cancer patients have a 3 times higher recurrence rate than non-cancer patients, making cancer one of the major risk factors for VTE (1, 2). Anticoagulant treatment is the cornerstone of the management of VTE at the cost of a non-negligible risk of bleeding. In this regard, reliable and validated clinical tools to predict thromboembolic and haemorrhagic events are crucial for individualized decision-making for the type and duration of anticoagulant treatment. Bleeding risk assessment models (RAMs) have been developed in unselected VTE patients under anticoagulant treatment, which have included a variable proportion of cancer patients. The *VTE-BLEED* and *RIETE* RAMs are currently the most widely used bleeding RAMs in common practice (3, 4). Multiple predictors are included in these RAMs, using both anticoagulation data (type, dosage and follow-up), patient characteristics (demography, genetics and comorbidities including cancer) and co-medications (5). Nevertheless, with the exception of the recently developed CAT-BLEED, no RAM has been developed to specifically predict the bleeding risk in cancer patients treated for VTE under anticoagulation (6). Indeed, of the 15 existing RAMs in the literature, the majority has limitations for cancer patients, including selection bias (patients included in phase III trials with an estimated minimum survival of one year at baseline) and variable follow-up ranging from 8 days to 3 years (7). In this context, it seems necessary to evaluate the available RAMs in real life cancer patients with VTE. The objective of the current study was to evaluate the performance of the different bleeding RAMs to predict the risk of bleeding in this population.

## 2. Methods

### 2.1. Selection of patients

For this observational retrospective cohort study, we selected patients with cancer and VTE from a pool of VTE patients, who were included prospectively and consecutively at the Louis Mourier Hospital (APHP, Colombes, France).

The inclusion criteria were the following: Patients with active cancer and a confirmed VTE, diagnosed at the Louis Mourier Hospital over a three-year period (between 01/01/2018 and 31/12/2020). All patients were followed over a 6-month period after VTE diagnosis. Cancer diagnosis was confirmed, either histologically (presence of a detectable tumor disease) or biologically (cancer biomarker). Additionally, patients whose cancer had been resected or not those who received antitumoral therapy, including hormonal therapy, within 6 months at the time of inclusion were also included in the study (8, 9). VTE comprised limb DVT or PE, and active cancer was defined as solid or haematological active cancer at the time of VTE (excluding skin cancers). Cancer is considered active when at least one of the following three conditions is met: (1) The patient received a potentially non-curative treatment for his cancer (in particular palliative chemotherapy); (2) The evolution shows that the treatment of cancer has not been curative (due to recurrence or progression under treatment) (especially recurrences after surgery); (3) Cancer treatment is underway.

VTE-diagnosed patient’s demographic and clinical characteristics, treatment regimens and outcomes for up to 6 months were collected.

The primary endpoint was the occurrence of a major bleeding (MB) or a clinically relevant non major bleeding (CRNMB) event, categorised according to the ISTH criteria (10, 11). According to the ISTH, an MB was defined as a fatal haemorrhage and/or symptomatic bleeding affecting a critical area or organ, such as intracranial, intramedullary, intraocular, retroperitoneal, intra-articular, or pericardial localization, or intramuscular with lodge syndrome, and/or bleeding responsible for a fall in haemoglobin of ≥20 g/L or resulting in the transfusion of more than 2 red blood cell units. According to the ISTH, CRNMB was defined as a bleeding other than an MB.

### 2.2. Authorization

All patients were included in accordance with the requirements of our hospital ethics committee. In our ethic charter, for data collected as part of individual patient follow-up no written consent is needed, other than the patient's welcome booklet of the hospital.

### 2.3. Selection of the risk assessment model

We performed a systematic search by keywords without time limitation (cancer associated thrombosis, VTE, DVT, PE, bleeding risk RAM, anticoagulant treatment) in PubMed, a bibliographic database of scientific articles (7). The fifteen bleeding RAMs available in the literature at the time of the study were the ACCP RAM, the Alonso et al. RAM, the CAT BLEED RAM, the Chopard et al. RAM, the EINSTEIN RAM, the HOKUSAI RAM, the KUIJER RAM, the Martinez et al. RAM, the NIEUWENHUIS RAM, the Nieto et al. RAM, the RIETE RAM, the two Skowrońska et al. RAM, the Seiler RAM and the VTE-BLEED RAM (3, 4, 6, 8, 12–21). We were able to gather predictors of 10 RAMs, and those predictors are presented in Table 1. For this work we could not assess the CATBLEED RAM, the EINSTEIN RAM and the HOKUSAI RAM, because no threshold is available in the literature at the time of the analysis (6, 14, 17). The two Skowrońska et al. RAM are derived from the RIETE RAM and the VTE BLEED RAM, obtained by adding a specific D-dimer level (19). Because D-dimer measurements were not systematically performed in our database, we were not able to utilize these RAMs. Among the 10 remaining RAMs, some RAMs presented missing predictors in our database, or not exactly defined as in our registry. When the predictor was not exactly defined as in our registry, we replaced this predictor with the closest predictor from our registry. No imputation methods were used in the analysis. Predictors included in the RAMs are presented in Table 2.

**Table 1:** Predictors included in anticoagulant RAM used in this study (3, 4, 8, 12, 13, 15, 16, 18, 20, 21).

	ACCP (8)	Alonso (12)	Chopard (13)	Kuijer (21)	Martinez (15)	Nieto (20)	Nieuwenhuis (16)	RIETE (3)	Seiler (18)	VTE-BLEED (4)
Demographic characteristics										
Age	X	X		X	X	X		X		X
Sex (Female or Male)		(F)		(F)	(M)					(M)
BMI					X		X			
Race										
Bleeding Risk factors										
Alcohol abuse	X	X				X				
History of bleeding	X	X	X		X	X	X	X	X	X
Kidney or/and Liver failure	X	X			X			X		X
Diabetes mellitus	X	X								
Uncontrolled hypertension. (±Male)										X
Recent surgical procedure	X						X			
Antiplatelet therapy and NSAIDs	X	X							X	
Poor anticoagulant control	X								X	
Frequent falls, previous stroke, dementia	X				X					
Recent traumatism					X		X			
Cancer history										
(active) Cancer or metastatic cancer	X	X		X	X	x		X	X	X
Genitourinary cancer										
Anticancer therapy with gastrointestinal toxicity										
Venous thrombosis Risk factors and History										
Pulmonary embolism as index event					X			X		
Distal DVT						X				
Other comorbidities										
Comorbidity + decrease of functional capacity / immobility	X					X			X	
Cardiovascular disease (Stroke/Coronaropathy / Peripheral arterial disease	X	X			X					
Syncope			X							
Tabaco and COPD		X			X					
Biological parameters										
Anemia/ Haemoglobin	X	X	x					X	X	X
INR/abdnormal prothrombin time	x					X			x	
Thrombopenia	X	X				x				X
D-Dimer										
Drugs										
Rivaroxaban		X								
Apixaban		X								
VKA		X								

**Table 2:** Predictors not available or modified for the study.

Score	Parameter	Data available in the registry	Alternative definition proposed by the authors	Risk biais in the score evaluation
ACCP RAM	NSAIDs	No	Data not available	Major = >2 data missing out of 19
	Poor anticoagulation control	No	Data not available	
	Comorbidity decrease of functional capacity			
Alonso et al. RAM	Alcohol absuse	No standard decision based on IC9 or ICD10	More than 4 drinks per day for men or more than 3 drins	Existing
	Renal disease			
	Chronic pulmonary disease			
	Liver disease			
	Anemia			
	Thrombocytopenia			
	Previous severe bleeding			
Martinez et al. RAM	History of bleeding or major	No time limit precised	Any bleeding event	Existing
	bleeding		Kidney failure stage	
	Renal dysfunction	No	New diagnosis defined as 90	
	New diagnosis of active cancer after	No time limit precised	Days prior or 90 days after the VTE event	
	VTE			
	Liver failure	No standard decision Defined from hospital	Any liver disease notified in the patient file	
	Dementia			
	Anemia	Discharge diagnoses and medical codes entered by GP	Any patient with a proof of a geriatric evaluation in his medical report	
	Cerebro vascular disease			
	Chronic pulmonary disease			
			Hb levels <130 g/L in males and <120 g/L in females within 182 days before the VTE	
			Any stroke or transient ischemic stroke	
Nieto et al. RAM	Abnormal prothrombine time	Cut off not specified	PT time <70%	Existing
	Renal dysfunction	Creatinine clearance <30 ml/min	Kidney failure stage	
Nieuwenhuis et al. RAM	Body surface area less than 2 m²	Formula used not specified	Data not available	Major = >1 data missing out of 5
Seiler et al. RAM	Low physical activity	No	Data not available	Major = >2 data missing out of 6
	Poor INR control	No	Data not available	
Skowrònska et al. RAM	D dimer levels >5750 ng/ml + RIETE or VTE BLEED	No	Data not available	Major this RAM si based on the additional biomarker value
VTE BLEED RAM	Anemia	Not defined	Hb levels <130 g/L in males and <120 g/L in females within 182 days before the VTE	Existing
	Renal dysfunction	No		
			Kidney failure stage	
	History of bleeding	No time limit precised	Any bleeding event	

For the ACCP RAM, 2 out of 19 predictors were missing. Despite that we were not able to calculate the exact scoring for the ACCP RAM, all the patients were classified at high risk. Therefore, we assumed that there was no impact of the missing predictors. For the NIEUWENHUIS RAM, the missing predictor is the body surface area under 2m2. The BMI denominator can be described as an area wrapped around a cylinder as tall as the body, and wide height/P. Therefore, according to Kurbel et al., the BMI denominator can be considered as a substitute for body surface area (22). We calculated the BMI denominator for all patients and none of them were under 2m2. Thus, with a few exceptions due to the limitation of the estimation of body surface area by the denominator of the BMI, this predictor accounts for zero points for all patients. For the SEILER RAM, poor INR control and the low physical activity status were missing as predictors (18). Regarding the poor INR control, we had no patients under VKA. The low physical activity was defined as following: “patient is either mostly sitting/lying and does not move a lot or often walks but avoids climbing stairs or to carry light weight <5 kg (self-report)” and this was considered by our team as not applicable and reliable in daily practice because of its subjectivity. Thus, the bias introduced did not interfere with our interpretation.

### 2.4. Analysis of the results

RAM overall discrimination performance was assessed through receiver operating characteristic (ROC) curves and concordance statistics values (representing the area under the ROC curve—AUC—with larger values indicating improved discrimination) were performed. To process the AUC analysis with C-statistics, we grouped all the scores with an obtained low risk of bleeding in one hand and the rest of the scores corresponding to an intermediate risk of bleeding or high risk of bleeding in the other hand. A C-statistics value over 0.8 was regarded a strong model. Additionally, a qualitative analysis was performed with the objective of displaying the treatment timeline before the bleeding event. Variables included for the analysis were: demographic characteristics (age, sex, BMI, race), bleeding risk factors (alcohol abuse, history of bleeding, kidney or/and liver failure, thrombocytopenia, diabetes mellitus, uncontrolled hypertension, recent surgical procedure, antiplatelet therapy, frequent falls, previous stroke, dementia, recent traumatism, genitourinary cancer, anticancer therapy with gastrointestinal toxicity), VTE risk factors and history [(active) cancer or metastatic cancer, PE as index event], others comorbidities (stroke, coronaropathy, peripheral arterial disease, tabaco use, chronic obstructive bronchopneumopathy) and biological parameters (anaemia/haemoglobin, thrombocytopenia).

Results were expressed with their correspondent 95% confidence intervals (95% CI). All statistical analysis was performed using IBM SPSS Statistics for Windows Version 26.0. (Armonk, NY: IBM Corp.). The TRIPOD checklist for validation of prediction models was used as recommendations for reporting prediction modelling studies in biomedical science (23).

## 3. Results

### 3.1. Patients baseline characteristics

During the study period, 110 patients with VTE and active cancer were included. Median age was 69.5 years old, with 38.2% of the patients being older than 75 years old. Our population was balanced gender wise, with 53.6% of the included patients being female. 79% of the population scored 2 or lower for an ECOG (Eastern Cooperative Oncology Group). The most frequent cancer sites were lung cancer (29.9%), followed by colorectal cancer (26.4%) and breast cancer (8.9%).

Renal failure was present in 23.6% of patients. Patient baseline characteristics, cancer characteristics and VTE characteristics are presented in Tables 3–5, respectively. The mean follow-up was 4.3 ± 2.3 months. 85 patients completed the follow-up, 47 patients died (death occurred within the 3 months following the VTE) and 3 were lost during follow up.

**Table 3:** Baseline characteristics of patients.

	Total (*N* = 110)
Age, years	69.5 ± 12.5
>75 years, no.(%)	42 (38.2)
Female, no (%)	59 (53.6)
Body-mass index, kg/m^2^^a^^,^^b^	25.7 ± 4.8
</=18 kg/m^2^, no (%)	6 (5.5)
>/=30 kg/m^2^, no (%)	21 (19.1)
World Health Organisation grade, no. (%)	
0	0 (0.0)
1	33 (30.0)
2	54 (49.1)
3	42 (38.2)
4	32 (29.1)
Bleeding medical history,no. (%)	
Renal failure	26 (23.6)
Previous bleeding	24 (21.8)
Alcohol abuse	15 (13.6)
Liver failure	12 (10.9)
Recent major bleeding (<1 month)	11 (10.0)
Major previous bleeding (<2 months)	8 (7.3)
Previous gastrointestinal bleeding (<10 days)	6 (5.5)
End stage renal failure	5 (4.5)
Previous gastrointestinal bleeding (>10 days)	4 (3.6)
Cardiovascular medical history, no. (%)	
History of hypertension	58 (52.7)
Diabetes	20 (18.2)
Tabacco use	19 (17.3)
Dyslipidemia	15 (13.6)
Blood pressure > 140 mmHg	7 (6.4)
Number of patient with cardiovascular risk factor	64 (58.2)
0	46 (41.8)
1	40 (36.4)
2	19 (17.3)
3	5 (4.5)
Peripheral arterial disease	10 (9.1)
Myocardial infarction	7 (6.4)
Ischemic stroke	5 (4.5)
Number of patient with cardiovascular disease	22 (20.0)
0	0 (0.0)
1	18 (16.4)
2	4 (3.6)
Other comorbid conditions, no. (%)	
Frequent falls, neurological or psychiatric disease	31 (28.2)
Atrial fibrillation	12 (10.9)
Chronic obstructive pulmonary disease	11 (10.0)
Recent trauma	7 (6.4)
Dementia	4 (3.6)
Drugs use	2 (1.8)
Valvular disease	0 (0.0)
Ongoing medication at the time of the inclusion, no. (%)	
Antiplatelet agents	20 (18.2)
Aspirin	17 (15.5)
Clopidogrel	3 (2.7)
Antidiabetics	18 (16.4)
Statins	12 (10.9)
Neuroleptics	11 (10.0)
Erythropoietin	3 (2.7)
CYP450 Inhibitors	0 (0.0)
Biology^b,c^	
Hemoglobin g/dl	11.4 ± 2.3
Platelets count 10.9/L	257.4 ± 111.5
PT time %	81.4 ± 19.8
White blood cells count 10.6/L	9.1 ± 4.8
Neutrophils count 10.6/L	6.6 ± 4.4
Créatinin mmol/L	93.3 ± 62.8
Protein C reactive mg/L	74.3 ± 68.2
ALT UI/L	35.9 ± 36.4
AST UI/L	36.1 ± 34.0
Total bilirubin UI/L	12.6 ± 6.9
GGT UI/L	129.3 ± 224.1
Albumin g/L	28.5 ± 6.9

^a^ Data were missing for 14 patients.

^b^ Plus-minus values are means ±standard déviation.

^c^ Data available for most of the patients.

**Table 4:** Cancer characteristics of the population.

	Total (*N* = 110)
History of malignancy, no (%)	11 (10.0)
Malignancy recurrence	10 (9.1)
Solid Tumor, no (%)	107 (97.3)
Type of solid tumor, no. (%)	
Lung	32 (29.9)
Colorectal	26 (24.3)
Breast	9 (8.4)
Prostate	8 (7.5)
Pancreas	6 (5.6)
Uterus	6 (5.6)
Gastric	5 (4.7)
Liver or biliary tract	5 (4.7)
ORL	4 (3.7)
Bladder or Urinary tract	3 (2.8)
Ovarian	2 (1.9)
Testis	1 (0.9)
Mestastasis, no. (%)	86 (80.4)
Brain metastasis	11 (10.3)
Treatment for the cancer, no. (%)	
Conventional chemotherapy	51 (46.4)
Surgical Tumor resection	30 (27.3)
Immunotherapy	16 (14.5)
Radiotherapy	10 (9.1)
Hormonotherapy	5 (4.5)
Biphosphonates	2 (1.8)
Targeted chemotherapy	1 (0.9)
Growth factors	1 (0.9)

**Table 5:** VTE index event characteristics of the population.

	Total (*N* = 110)
History of VTE, no (%)	13 (11.8)
Pulmonary Embolism	3 (2.7)
Lower limb DVT	5 (4.5)
Upper limb DVT or atypical site DVT	4 (3.6)
Superficial venous thrombosis	1 (0.9)
Risk factor of VTE, no (%)	29 (26.4)
At least one unrpovoked VTE	0 (0)
Presence of a vena cava filter	0 (0)
Surgery in the last 3 month	7 (6.4)
Immobilisation	20 (18.2)
Major thrombophilia	1 (0.9)
Minor thrombophila	0 (0)
Hormonal tratement	1 (0.9)
Number of patient with VTE risk factor	
0	0 (0)
1	27 (24.5)
2	2 (1.8)
Delay between the diagnosis of cancer and the diagnosis of the VTE index event (month)	15.5 ± 24.5
Asymptomatic presentation of the VTE, no (%)	28 (25.5)
Asymptomatic PE	18 (16.4)
Asymptomatic limb DVT	8 (7.3)
Pumonary embolism localisation, no (%)	78 (70.9)
Sub segmental distal PE	4 (3.6)
Proximal PE	74 (67.3)
DVT, localisation, no (%)	47 (42.7)
Proximal lower limb DVT	24 (21.8)
Distal lower limb DVT	8 (7.3)
Proximal and distal component lower limb DVT	4 (3.6)
Catheter related upper limb DVT	11 (10.0)
DVT and PE association, no (%)	21 (19.1)

VTE, venous thromboembolism; DVT, deep venous thrombosis; PE, pulmonary embolism.

During the 6 months follow-up period, 26 out of 110 included patients (26.7%) experienced a bleeding event, with 3 recurrences of bleeding. Out of the 29 bleeding events, 19 events were CRNMB and 10 MB. One patient died because of a fatal bleeding just after the anticoagulation introduction. 3 more patients died while bleeding, leading to a fatal bleeding related rate of 4.5%. Most bleeding events occurred in the first 3 months of the follow up: 81% of the MB and 78% of the CRNMB. Considering the first bleeding event, 54% (14/26) occurred during the first month. Most of the bleedings were gastrointestinal (9 events, 31%); 26.9% of the bleedings occurred in patient with colorectal cancer and 19.6% in patients with lung cancer. The type and site of the bleeding events are presented in Table 6.

**Table 6:** Types and sites of bleedings the population.

	Total (*N* = 26)
Occult bleeding, no (%)	2 (8)
Gastric cancer	1 (4)
ENT cancer	1 (4)
Spontenaous bleeding, no (%)	6 (23)
Epistaxis	1 (4)
Lung cancer	1 (4)
Hemoptysia	1 (4)
Lung cancer	1 (4)
Brain localisation	2 (8)
Pancreas cancer	2 (8)
Hematoma	4 (16)
Brest cancer	1 (4)
Colo-rectal cancer	2 (8)
Uterus cancer	1 (4)
Hematuria	1 (4)
Prostate	1 (4)
Digestive tract	9 (35)
High digestive tract	2 (8)
Gastric cancer	1 (4)
ENT cancer	1 (4)
Low digestive tract	7 (27)
Colo-rectal cancer	3 (12)
Pancreas cancer	1 (4)
Lung cancer	1 (4)
Breast cancer	1 (4)
Urothelial cancer	1 (4)
Post traumatic bleeding, no (%)	6 (24)
Colo-rectal cancer	3 (12)
Lung cancer	2 (8)
Breast cancer	1 (4)

Regarding the risk of VTE recurrence, 5 patients, including 3 who bled previously, presented a VTE recurrence (DVT or atypical site DVT). One patient died from VTE from the initial event and one patient died from a VTE recurrence. Leading to a fatal VTE related rate of 1.8%.

### 3.2. Qualitative assessment of the bleeding event

**Figure 1:**
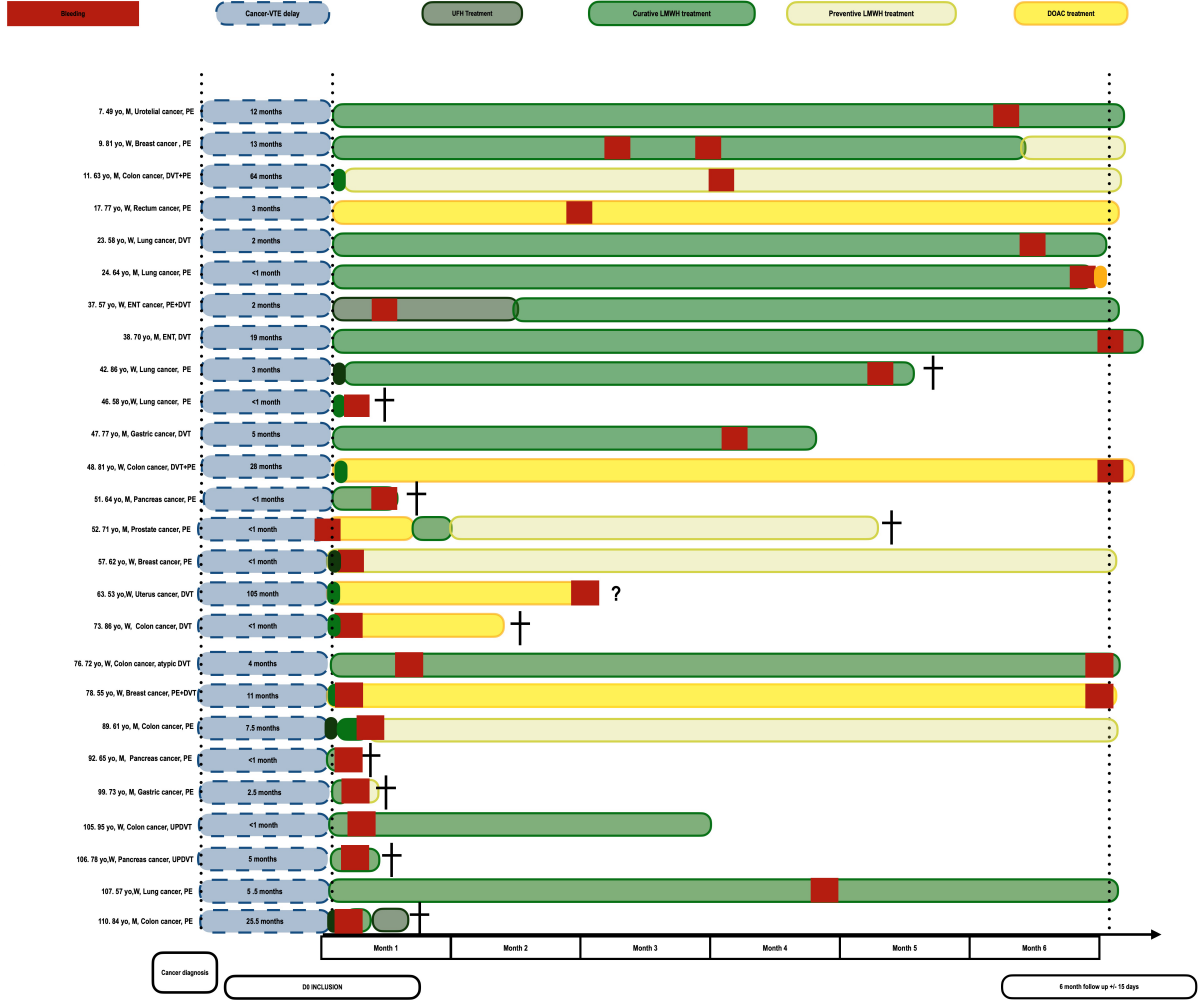
Treatment timeline of patient with bleeding events.

Overall, 84.6% (22/26) of first bleeding events occurred in patients treated with LMWH or unfractionated heparin, which represents 22% of the patients treated with these drugs at the inclusion (22/98). Fifteen bleeding events with LMWH or unfractionated heparin occurred during the 3 first months (68.2%, Figure 1).

Regarding the association between the use of DOAC and bleeding events, 2 out of the 6 patients that were prescribed DOAC as the first treatment for the thrombotic events bled. Bleedings occurred in 3 out of the 20 patients that were prescribed DOAC as the second treatment. All the patients that displayed with bleedings under DOAC had non removed colorectal or uterus cancer and 4 bled from their cancer site (Figure 1). The rate of bleeding (27%) was more important in the group of patient treating with DOAC that in the patient treated with LWMH (22%) with a non signififcative difference. Moreover, 2 patients with digestive cancer bled under each of the treatment.

Eight patients among the 26 patients that experienced bleeding had a renal impairment (<60 ml/min of renal clearance) with a mean renal clearance at 72 ml/min without difference with the mean clearance of patients that did not bleed (74 ml/min). The two patients of our cohort that had thrombopenia below 75,000 platelets per liter, respectively 25,000 and 49,000 platelets per liter were treated with unfractioned heparin at prophylactic dose and did not experience bleeding.

### 3.3. Distribution of patients according to different RAMs and RAM performance analysis

Whether the patient bled or not, according to the ACCP RAM and the VTE BLEED RAM all patients would be classified in the high-risk category. Whether the patient bled or not, the NIEUWENHUIS RAM classified most of the patients (85 out of 110) in the low-risk and no patients in the high-risk category. 24 out of the 26 patients that bled were classified as low risk.

Among the patient with bleedings, the Chopard et al. RAM (16/26), the KUIJER RAM (24/26) and the Martinez et al. RAM (22/26) classified most patients in the high risk category. The Alonso et al. RAM (15/26), the Nieto et al. RAM (13/26), the Seiler et al. RAM (13/26) and the RIETE RAM (17/26) classified most bleeding patients in the intermediate risk category. No patients with bleedings were classified in the low risk category for the Alonso et al. RAM, the KUIJER RAM and the RIETE RAM.

**Figure 2:**
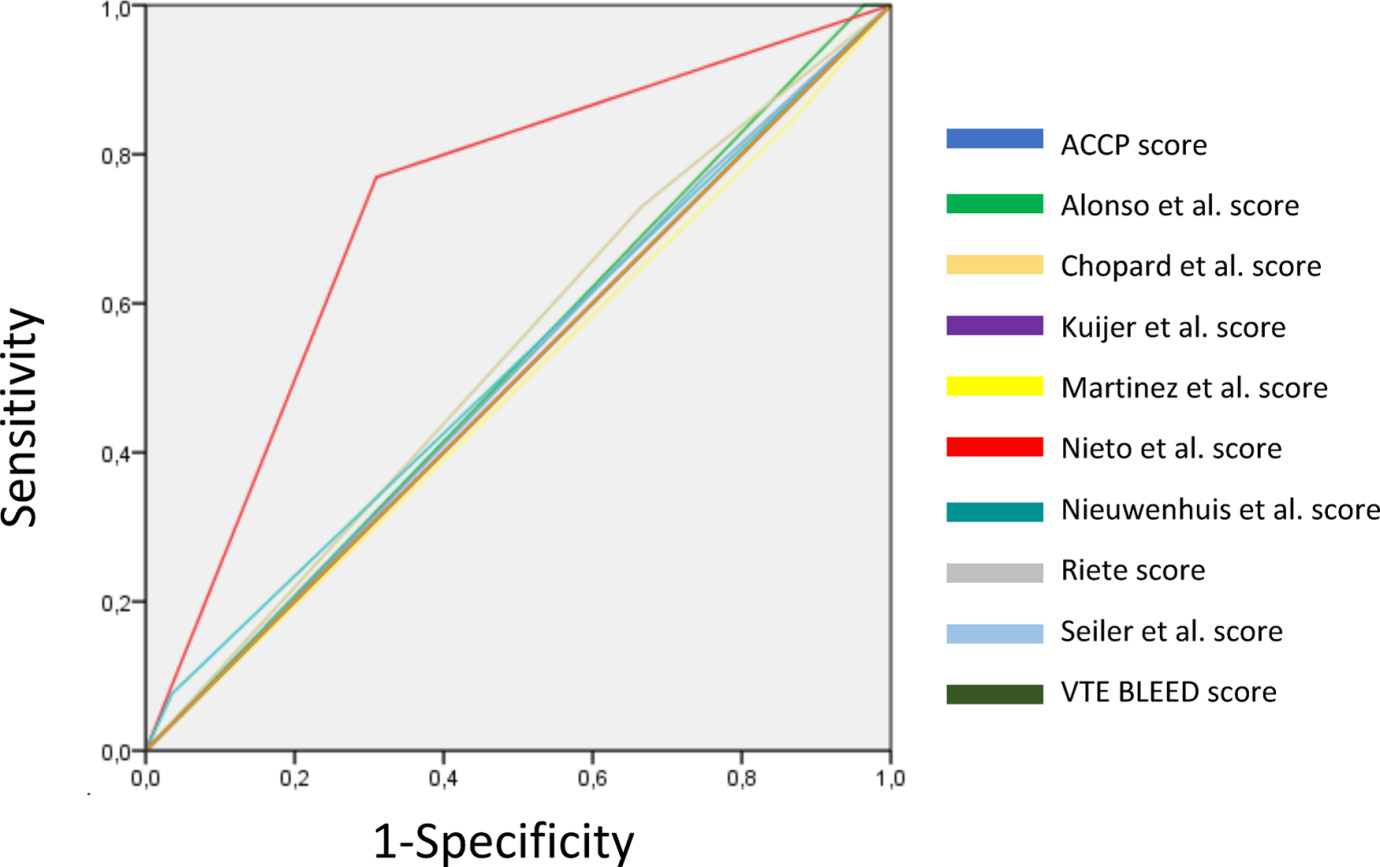
ROC curves of the different RAMs.

Among individuals without bleedings, no RAM classified patients in the low-risk category. When analysing the ROC curves and C-statistics, the Nieto et al. RAM had the best C-statistics result. These results are summarized in Tables 7, 8 and Figure 2.

**Table 7:** Concordance statistic of the different RAMs.

	C-stats (95% CI)	Inferior limit 95% CI	Superior limit 95% CI
ACCP RAM	0.500	0.372	0.628
ALONSO RAM	0.518	0.393	0.643
CHOPARD RAM	0.532	0.406	0.658
KUIJER RAM	0.500	0.372	0.628
MARTINEZ RAM	0.489	0.360	0.617
NIETO RAM	0.730	0.619	0.840
NIEUWENHUIS RAM	0.521	0.391	0.650
RIETE RAM	0.500	0.372	0.628
SEILER RAM	0.510	0.383	0.636
VTE BLEED RAM	0.500	0.372	0.628

RAM, risk assessment model.

**Table 8:** Distribution of patients according to different RAMs.

Risk	ACCP	ALONSO	CHOPARD	KUIJER	MARTINEZ	NIETO	NIEUWENHUIS	RIETE	SEILER	VTE BLEED
High (Bleeding+/Bleeding-)	25/84	11/43	16/16	25/74	21/59	7/26	0/0	9/32	7/4	26/84
Intermed (Bleeding+/Bleeding-)	1/0	15/38	3/40	1/10	/	13/43	2/23	15/52	19/59	0/0
Low (Bleeding+/Bleeding-)	0/0	0/3	7/28	0/0	4/25	6/13	24/61	0/0	0/21	0/0

## 4. Discussion

In our cohort, none of the ten RAMs used in our study were able to distinguish cancer patients with a low risk of bleeding, from all bleeding or non-bleeding patients. The Nieto et al. RAM had the best overall performance [C-statistic = 0.730, 95% CI (0.619–0.840)] (20). However, it classified 5 patients with major bleeding (5 out of 26) in the low risk of bleeding group. The rest of the RAMs showed a suboptimal result, with a range of C-statistic between 0.489, 95%CI (0.360–0.617)) for the Martinez et al. RAM and 0.532, 95%CI (0.406–0.658) for the Chopard et al. (13, 15). The Chopard et al. RAM (7/26), the Martinez et al. RAM (4/26), the NIEUWENHUIS RAM (24/26), the Nieto et al. RAM (6/26) and the Seiler RAM (6/26) classified bleeding patients in the group of low risk of bleeding.

In our population of patients with cancer and VTE, the distribution between cancer sites is comparable to the distribution found in observational studies. However, we registered a higher proportion of bleeding events (26.7% in our study vs. approximatively 12.5% in other studies) (24). This might be explained by the fact that our population is older (38.2% of the patients is older than 75 years old vs. 30.2% in other studies) and with more comorbidities (x% vs. x%). There is an increased need for individualized decision making for cancer management. In our previously published review, we deemed it necessary to answer the questions how and when to assess anticoagulant-associated bleeding risk, as well as what outcome to assess for which patients (7).

The CAT patients for whom we assess the anticoagulant-associated bleeding risk have different characteristics as compared to cancer-free patients with VTE, and differences exist also among CAT patient (24). All the patients of our cohort were classified at intermediate or high risk of bleeding by available RAMs.

Including cancer-specific variables in the RAMs, and possibly even site-specific variables, might help improve their performance in cancer patients. It is possible that CAT risk assessment will be improved either by the development of RAMs specific to cancer sites or by modifications of existing RAM that incorporate site-specific CAT risk factors, especially for colorectal cancer patients of whom one third will develop bleeding and which represents the most VTE-associated cancer, after lung cancer.

It is important to define how the anticoagulant-associated bleeding risk in CAT patients is assessed and whether differences should be taken into account rather than an overall assessment. No RAM takes into consideration important aspects related to bleeding and thrombosis in cancer patients, such as drug-drug interaction and consequences of therapy such as chemotherapy (especially gastrointestinal), the use of growth factors or radiotherapy, or the use of other supportive therapies like catheter implantation or transfusions (25). Moreover, among the RAMs tested only the Alonso et al. RAM proposed to add points in their RAM depending on the type of anticoagulant of the patients (DOAC or not DOAC). We argue that the risk might differ when patients are treated with DOAC or heparins (12). It is necessary to propose tools that suit the treatment and evolve with care, and in subject of study, to propose specific RAMs according to the anticoagulant treatment.

What is evaluated as the principal outcome by most of the RAMs is MB. The ACCP RAM and VTE-BLEED RAM classified all the patients included in our study in the high risk of bleeding category. Regarding major bleeding events, the ACCP RAM, the KUIJER et al. RAM, the Martinez et al. RAM and the VTE-BLEED RAM classified all the patients with MB events in the high risk of bleeding group (4, 8, 15, 21). One patient died from an MB and this individual was classified at least in the intermediate risk of bleeding group for all the RAMS but the NIEUWENHUIS et al. RAM, which classified the patient in the low risk of bleeding group (16). Of note, the NIETO RAM was developed to predict of the risk of anticoagulant-associated fatal bleeding risk, and this RAM classified the patient in the group of patients at high risk of death from fatal bleeding (20). Regarding CRNMBs, only the ACCP RAM and VTE-BLEED RAM classified all patients with CRNMB events in the high risk of bleeding group (4).

Among the first bleeding events, 54% (14/26) of the bleedings (MB and CRMB) occurred during the first month and 90% (9/10) of the MBs occurred during the 3 first months. No international recommendation supports a treatment for VTE of less than 3 months, so it might be too early to assess the risk of bleeding at that time even if it is the time when most of the bleeding events occurred. A RAM for anticoagulant-associated bleeding in the setting of the prevention of recurrent VTE should ideally be targeted at patients who have already completed the initial length of treatment.

To our knowledge, there is no published study evaluating the performance of RAMs in patients with CAT using real world patients. Recently, Sanfilippo et al. presented at a study using 7,489 patients with CAT (mean age of 66.9 years old) assessing the CAT-BLEED RAM, the VTE BLEED RAM and 3 other RAMs developed for atrial fibrillation patients (26). Their conclusion was that the different RAMs demonstrated a poor predictive performance for MBs, reflecting the difficulty to predict the occurrence of MB in cancer patients regardless of the indication of anticoagulant.

The current study presents several limitations. It was performed on a database whose original purpose was not to evaluate RAMs. However, most RAM development and validation studies are performed in similar conditions and our cohort presents the advantage of having thrombotic events as the primary endpoint, consecutive non-selected patients and prospective collection of data. The available data allowed us to compute scores of all patients.

Within this study we ought to assess which RAM can isolate patients at risk of bleeding and who will benefit from personalized anticoagulant treatment. Unfortunately, the CAT-BLEED RAM, the EINSTEIN RAM, the HOKUSAI RAM and the two SKOWROŃSKA et al. were not be tested on our patient population (6, 14, 17, 19). For the CAT-BLEED RAM, the EINSTEIN RAM, the HOKUSAI RAM no accessible threshold was published when we performed the study. For the Skowrońska et al. RAM, derived from the RIETE RAM and the VTE BLEED RAM, we can say that for no patient the estimation of the risk will be low despite the absence of the D dimer data. As shown in Table 2, three other RAMs (ACCP RAM, NIEUWENHUIS RAM and the Seiler et al. RAM), presented risk of bias in the evaluation but were still included.

The biggest flaw of the study is its limitation in the sample size of the patients. From a statistical point of view, our study is not able to validate any of the RAMs. Despite this weakness, the current study is one of the first works testing anticoagulant-associated bleeding RAMs in cancer with real world data and our qualitative assessment of treatment timeline is a new method to display the relation between bleeding events and anticoagulant treatment. Moreover, our work is in agreement with previous work that can be found in the literature on populations of cancer patients from randomized controlled clinical trials.

## 5. Conclusion

The management of CAT patients is challenging due to a higher risk of both recurrent VTE and bleeding events, as compared with non-cancer patients with VTE. None of the existing RAMs was able to consistently identify patients with risk of anticoagulant associated bleeding events. This study displays a practical illustration of the comments made in our previous review (7). Optimization of the assessment of bleeding risk in CAT patients needs to be undertaken.

## Data Availability

The raw data supporting the conclusions of this article will be made available by the authors, without undue reservation.
